# Hemorrhagic complications following open surgical biopsy in pediatric liver tumors: 3 cases, a review of literature and a plea for image-guided core needle biopsy

**DOI:** 10.3389/fped.2026.1971804

**Published:** 2026-10-29

**Authors:** Julian Kolorz, Jan Sabo, Maren Schulze, Sebastian Fritzemeier, Michael M. Schündeln, Simone Kathemann, Elke Lainka, Lars Pape, Michael Berger

**Affiliations:** 1 Department of Pediatric Surgery, University Hospital Essen, University of Duisburg-Essen, Essen, Germany; 2 Department of General, Visceral- and Transplant Surgery, University Hospital Essen, University of Duisburg-Essen, Essen, Germany; 3 Department of Pediatrics I, Neonatology, Pediatric Intensive Care, Pediatric Neurology, University Hospital Essen, University of Duisburg-Essen, Essen, Germany; 4 Division of Pediatric Hematology and Oncology, Department of Pediatrics III, University Hospital Essen, University of Duisburg-Essen, Essen, Germany; 5 Division of Pediatric Gastroenterology and Hepatology, Department of Pediatrics II, University Hospital Essen, University of Duisburg-Essen, Essen, Germany; 6 Department of Pediatric Surgery, St. Elisabeth Hospital Essen, Essen, Germany

**Keywords:** core needle biopsy, hemorrhage, hepatoblastoma, liver biopsy, pediatric liver tumor

## Abstract

**Background:**

Histopathological confirmation remains an important component of the diagnostic workup of pediatric liver tumors. Despite the growing consensus that image-guided core needle biopsy is the least traumatic strategy, open surgical biopsy or intraoperative tumor manipulation is still performed in a selected group of patients. The potential complications associated with surgical manipulation of pediatric liver tumors may be underappreciated in current diagnostic algorithms.

**Methods:**

Here, we report three consecutive cases of children referred to our tertiary pediatric liver center following severe hemorrhagic complications and requiring intensive care management after open surgical biopsy or manipulation. A narrative review of the literature regarding pediatric liver tumor biopsy was performed, focusing on indications, technical considerations, periinterventional management, and rescue management.

**Results:**

All three children developed severe hemorrhagic complications following open surgical biopsy or manipulation of a liver tumor. Complications included hemorrhage and coagulopathy, which required abdominal packing, temporary abdominal wall non-closure, mechanical ventilation and intensive care treatment, sequentially leading to referral to our pediatric liver center. One child developed hemorrhagic shock with acute liver and kidney failure and required high-urgency liver transplantation for hepatoblastoma. Final diagnoses were hepatoblastoma, hepatocellular neoplasm not otherwise specified (HCN-NOS) and malignant rhabdoid tumor of the liver.

**Conclusion:**

Open surgical biopsy of pediatric liver tumors should not be regarded as a minor diagnostic procedure. If feasible, biopsy should be carefully planned in a multidisciplinary setting and performed as image-guided core needle biopsy with coaxial technique, controlled pediatric anesthesia and a predefined hemorrhage rescue pathway. Avoiding open biopsy whenever feasible may significantly improve patient safety.

## Introduction

Pediatric liver tumors are rare childhood malignancies accounting for 1%–2% of all pediatric malignancies and require highly specialized multidisciplinary care (1, 2). Hepatoblastoma (HB) is the most common primary malignant liver tumor in childhood and typically affects infants and young children (3, 4). Management of HB relies on high-quality imaging, histopathological classification, serum alpha-fetoprotein (AFP), risk stratification, chemotherapy, resection, and, in selected patients, liver transplantation (LT) (3, 5–8). Histopathological confirmation remains necessary in most children with suspected malignant liver tumors, especially when upfront complete resection is not appropriate or when tumor biology is uncertain (2). Tumor tissue is required to establish the diagnosis and furthermore, to distinguish HB from other liver malignancies such as malignant rhabdoid tumor (MRT), hepatocellular carcinoma (HCC), metastatic disease, vascular tumors, or other rare hepatic lesions.

One central issue is not whether tissue can be obtained, but how to do so safely and effectively. Historically, open surgical biopsy was frequently performed; however, advances in pediatric surgery and interventional radiology now allow for precise image-guided core needle biopsy with high diagnostic yield and minimal tissue trauma (9–17).

Consequently, in many situations of HB it has become standard of care to not perform a biopsy at all. In children with typical age, elevated AFP, and characteristic imaging findings, hepatoblastoma may be diagnosed clinically without biopsy (18). In contrast, histological confirmation was generally required within the PHITT protocol (19). When biopsy is required, image-guided percutaneous biopsy provides high diagnostic yield with low morbidity (14).

Despite this evidence, open biopsy and unplanned intraoperative manipulation of pediatric liver tumors still occur. Such procedures can be dangerous because large pediatric liver tumors are often hypervascular, fragile, and located near major vascular structures. In infants and small children, even moderate blood loss may lead to hemodynamic instability, coagulopathy, abdominal compartment syndrome, or even organ failure.

Recently, our tertiary pediatric liver center received three consecutive children with severe hemorrhagic complications after open surgical biopsy. We present these cases and discuss the evidence supporting image-guided core needle biopsy as the preferred diagnostic strategy whenever feasible.

## Case series

Patient characteristics for all three cases are listed in Table 1.

**Table 1:** Patient characteristics of cases 1–3.

Case	Age	Weight	Gender	Initial presentation	Tumor suspicion/Imaging	Initial procedure	Complications	Rescue management	Final diagnosis	Outcome
1	15 months	11 kg	M	Progressive abdominal distension, fatigue, night sweats, anemia	MRI with heterogenous cystic liver tumor, pulmonary metastases	Open biopsy	Severe bleeding, massive liver swelling, coagulopathy	Transfer intubated to pediatric liver center, transfusion of 6 PRBCs, FFP and platelets	MRT of the liver	ICU stay, re-exploration, delayed closure of abdomen, chemotherapy after stabilization
2	7 years	17 kg	M	Acute abdominal pain, suspected appendicitis	Unexpected tumor, ruptured necrotic lesion in segment VII	Diagnostic laparoscopy, conversion to laparotomy, exploration of tumor	Tumor rupture, hemoperitoneum	Packing and transfer intubated to pediatric liver center, transfusion of 2 PRBCs	HCN-NOS	ICU stay, second-look and needle core biopsy, chemotherapy after stabilization
3	18 months	12 kg	M	Severe abdominal pain, firm distended abdomen	High AFP, MRI suspicious for HB	Open biopsy	Severe bleeding, abdominal compartment, hemorrhagic shock, acute liver and kidney failure	Packing and transfer intubated to pediatric liver center, transfusion of 2 PRBCs	HB	ICU stay, hemodialysis, HU listing, urgent split-LT, chemotherapy after stabilization

M, male; MRI, magnetic resonance imaging; AFP, alpha-fetoprotein; HB, hepatoblastoma; PRBCs, packed red blood cells; FFP, fresh frozen plasma; HCN-NOS, hepatocellular neoplasm not otherwise specified; MRT, malignant rhabdoid tumor; ICU, intensive care unit; HU, high-urgency; LT, liver transplantation.

### Case 1

A 15-month-old child was referred to our tertiary pediatric liver center after a complicated open biopsy of a large intrahepatic tumor. According to the referral history, the child had developed progressive abdominal distension over approximately 6 weeks, accompanied by fatigue and night sweats. Initial evaluation at the local pediatrician demonstrated leukocytosis and anemia. Ultrasound (US) revealed a large hepatic mass and magnetic resonance imaging (MRI) showed a heterogeneous, partially cystic liver tumor with an estimated volume of approximately 800 mL and multiple pulmonary metastases. Because tumor dignity remained unclear, open surgical biopsy was performed at the referring institution. During the procedure, severe bleeding occurred together with marked swelling of the liver and progressive laboratory evidence of coagulopathy. Primary abdominal wall closure was not possible. Six units of packed red blood cells (PRBCs), fresh frozen plasma and platelets were transfused, and the child was urgently transferred on mechanical ventilation for further management. At re-exploration, there was no active bleeding, but the tumor was large and the hepatic tissue fragile. After repositioning of the bowel, delayed abdominal wall closure was achieved, and a sterile vacuum-assisted dressing was applied. Reference pathology subsequently established the diagnosis of MRT of the liver. After stabilization, systemic chemotherapy was initiated.

### Case 2

A 7-year-old child presented to a local hospital with acute abdominal pain and was initially suspected to have acute appendicitis. Diagnostic laparoscopy revealed a small amount of intraperitoneal blood and an unexpected liver tumor. The procedure was converted to laparotomy, and the liver tumor was biopsied. Active bleeding from the biopsy site developed and hemostasis could only be achieved by abdominal packing with surgical towels. Two units of PRBCs were transfused, and again the patient was transferred intubated and mechanically ventilated by helicopter to our center. On arrival, the child was cardiopulmonary stable. During a planned second-look laparotomy, no new intraperitoneal bleeding was identified. After careful depacking, the site of tumor rupture could be identified. The tumor was located in segment VII and appeared necrotic and ruptured. MRI showed further lesions in both liver lobes. Staging was PRETEXT III (annotation factors R+, F+, P−, V−, E−) without pulmonary metastases. Definitive hemostasis was achieved and sufficient tumor material was obtained by core needle biopsy. A drain was placed behind the liver, and definitive abdominal closure was performed. Histopathology initially suggested a partially fetal HB with hepatocellular components. The final diagnosis was hepatocellular neoplasm not otherwise specified (HCN-NOS) with a TERT promoter mutation. Preoperative AFP was 159,393 IU/mL and decreased to 58,689 IU/mL on follow-up measurement. The child remained stable after extubation and proceeded to further oncological management.

### Case 3

An 18-month-old child was transferred to our pediatric intensive care unit after acute bleeding following an open liver biopsy performed for suspected HB. The child had presented 1 week earlier with severe abdominal pain and a distended abdomen. US and computed tomography raised suspicion of HB. AFP was greater than 150,000 IU/mL, and MRI was also considered highly suspicious for HB. The tumor was PRETEXT IV and thus not amenable to upfront resection. The annotation factors were E+, R+ and V+ (tumor board after transplantation). Pulmonary metastases were not definitely visible at presentation (M0, assessment limited by atelectasis), but a CT 13 days after transplantation showed pulmonary metastases (M1, high-risk group). At the referring institution, Broviac catheter implantation and open liver biopsy were performed. The procedure was complicated by diffuse bleeding. Re-operation was performed because of suspected abdominal compartment syndrome, but diffuse bleeding could not be controlled. Abdominal packing and abdominal wall closure were performed. Two units of PRBCs were transfused. The next day after transfer to our center, re-laparotomy with placement of a vacuum-assisted closure (VAC) system was performed. At the time of transfer, the child had persistent anuria, rising retention parameters, and worsening liver function. Because of hemorrhagic shock, the patient developed acute liver failure and kidney failure, requiring hemodialysis and rendering planned neoadjuvant chemotherapy impossible. After interdisciplinary discussion, the patient was evaluated for liver transplantation and subsequently listed with high urgency. Following availability of a suitable deceased donor organ, a left lateral split graft (segments II/III) was transplanted. The explanted liver (1,066 g) contained a large embryonal HB with capsular breakthrough, portal vein infiltration (R1) and a peritoneal tumor nodule. Intraoperatively, severe coagulopathy required transfusion of three PRBCs, fresh frozen plasma, platelets, fibrinogen, prothrombin complex concentrate, and tranexamic acid. Postoperatively, graft function improved rapidly, hepatic flows remained patent, and liver enzymes and cholestasis parameters decreased. The postoperative course was complicated by hemothorax, septic shock and a biloma requiring revision of the biliary anastomosis. Chemotherapy (doxorubicin and cisplatin, with cisplatin initially reduced by 50% because of dialysis) was started. Two months after transplantation, dialysis was no longer required and MRI showed no definite pulmonary metastases.

## Discussion

The three cases show a clinically important problem in pediatric liver tumor diagnostics: biopsy itself can become the source of major complications. Although the clinical scenarios differed, all cases shared a similar trajectory. A large pediatric liver tumor was surgically manipulated or biopsied through an open approach, severe bleeding followed, and tertiary liver-center rescue management became necessary. The hemorrhage required packing, temporary abdominal wall non-closure and transfusion of PRBCs and, in one case, led to acute liver and kidney failure.

Histopathological confirmation remains important in many pediatric liver tumors, particularly when the diagnosis is uncertain or tissue is required for molecular characterization. However, biopsy is not universally required in children with a typical presentation of HB. In selected children aged 6 months to 3 years with characteristic imaging findings and markedly elevated AFP, treatment may be initiated without histological confirmation, whereas histological confirmation was generally required in the PHITT protocol or when liver transplantation is being considered (18, 19). The first case in our series highlights this dilemma. Despite a large hepatic mass in an infant, reference pathology ultimately demonstrated MRT rather than HB. However, the need for tissue does not imply the need for open biopsy. The strongest available pediatric evidence is the COG AHEP0731 analysis of 121 children with initially unresectable HB undergoing biopsy at diagnosis. All biopsy approaches yielded adequate tissue. In contrast, clinically significant post-biopsy hemorrhage requiring transfusion occurred in 36% after open biopsy (*n* = 27/76), 24% after laparoscopic biopsy (*n* = 4/17), and 0% after percutaneous biopsy (*n* = 0/28) (17). In another systematic review by the APSA Cancer Committee, 307 children with liver tumors were included, most commonly with HB. Compared with surgical biopsy, core needle biopsy was associated with markedly lower overall morbidity and lower bleeding risk, while maintaining a high diagnostic yield (20). More recent HB-specific data further support this approach, such as the publication by Schoeman et al. reported high diagnostic adequacy, reliable histological subtyping and successful molecular profiling after image-guided percutaneous biopsy, without hemoperitoneum (14). Pediatric hepatology guidance, such as the position paper of the ESPGHAN committee, likewise recommends ultrasound- or CT-guided biopsy when focal liver lesions require sampling, while reserving plugged-tract, transjugular, laparoscopic, or open approaches for selected situations in which standard percutaneous access is unsafe or inadequate (10). Percutaneous biopsy is not risk-free, and our three cases cannot show that needle biopsy would have ultimately avoided these events. However, these findings collectively support the avoidance of open surgical biopsy when image-guided percutaneous biopsy is feasible.

There are several reasons why open biopsy or surgical manipulation of pediatric liver tumors may be hazardous (15, 17). First, HBs and other malignant liver tumors can be hypervascular and fragile. Second, large tumors frequently alter vascular anatomy, stretch the capsule or contain necrotic areas. Third, infants and toddlers have limited blood volume. Blood loss that may appear moderate in an adult can be catastrophic in a small child. Fourth, open surgical exposure may disrupt the capsule or necrotic tumor surface and create a bleeding surface that lacks the tamponade effect of a narrow transhepatic needle tract. These mechanisms were all reflected in our cases. Case 1 developed massive liver swelling and coagulopathy after open biopsy of a large metastatic liver tumor. Case 2 demonstrates the danger of unplanned tumor manipulation. Diagnostic laparoscopy for presumed appendicitis revealed hemoperitoneum and an unexpected tumor, followed by conversion to laparotomy and packing. Case 3 represents the most severe consequence, with diffuse bleeding after open biopsy progressing to shock, renal failure and liver failure.

In all three cases, the indication and choice of biopsy approach were discussed with the referring team. In Cases 1 and 2, the responsible surgeons reported that the diagnostic potential of image-guided core needle biopsy and the risks associated with open surgical biopsy had not been fully appreciated at the time of the initial intervention. In Case 3, the decision to obtain tissue was made jointly by the responsible surgeon and the pediatric oncologist, despite the patient's young age, markedly elevated AFP, and imaging findings highly suggestive of hepatoblastoma.

In Case 1, tissue diagnosis was necessary (MRT) and core needle biopsy would have been appropriate. In Case 2, hemoperitoneum was already present and the tumor was ruptured, so the bleeding cannot be attributed to the biopsy alone, and percutaneous biopsy of a ruptured tumor may itself be hazardous; stabilization and imaging should take priority (Figure 1). Case 3 raises the question whether biopsy was needed at all: with an age of 18 months, AFP >150,000 IU/mL, typical imaging and PRETEXT IV, treatment could have been started without histology (18). In this case, the decision to obtain tissue was made jointly with the pediatric oncologists, who requested histological confirmation.

**Figure 1:**
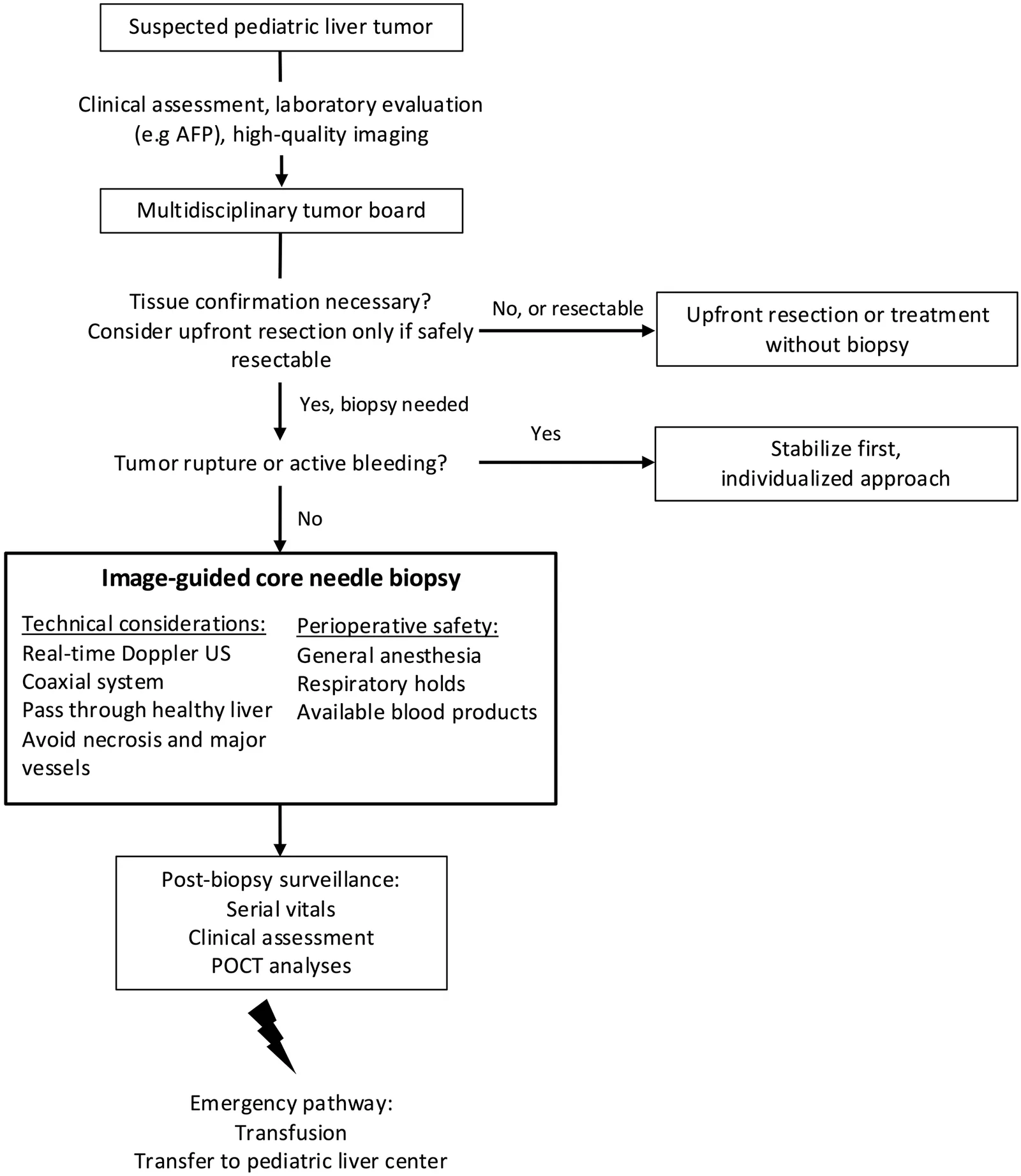
Proposed safety algorithm for pediatric liver tumor biopsy.

Image-guided core needle biopsy offers several advantages over open surgical biopsy (11, 13, 14, 20). It allows precise targeting of viable tumor tissue, while avoiding major vascular structures under ultrasound guidance. In contrast to open biopsy, it avoids unnecessary surgical manipulation of the tumor, reduces morbidity, and may allow faster recovery with oncologic treatment to start without delay. A critical technical principle is trajectory planning. Whenever feasible, the biopsy needle should pass through healthy liver parenchyma before entering the tumor rather than directly puncturing a subcapsular tumor surface. This transhepatic approach may provide a tamponade effect along the needle tract and reduces the risk of free intraperitoneal bleeding. The target should be viable tumor tissue at the tumor-parenchyma interface, avoiding both large necrotic cavities and major vascular structures. Doppler ultrasound should be used systematically to map vascular anatomy before tissue acquisition. Coaxial biopsy systems are particularly attractive because they allow multiple cores through a single capsular puncture, which may reduce repeated capsular trauma (11, 13, 20).

If doppler ultrasound cannot reliably assure safe or precise biopsy of the designated area, needle biopsy can be carried out under laparoscopic guidance. At our center, this is standard procedure and implies placing a 5 mm trocar in the umbilicus for the camera as well as one or two additional 3 mm working ports for retraction or, more importantly, for coagulation. The percutaneous needle is then inserted separately, and the core needle biopsy is then taken under direct laparoscopic visualization. If needed, immediate coagulation to the area of the biopsy can be applied laparoscopically with the bipolar instruments.

In this same sense, liver tumor biopsy in children should always be considered a high-risk procedure. It should be carefully planned and not performed as a minor bedside procedure. General anesthesia with endotracheal intubation can aid biopsy due to immobility of the patient, analgesia and continuous hemodynamic monitoring. Another procedural pearl is controlled ventilation of the patient. The anesthesiologist can perform a respiratory hold, if needed, during biopsy. Such maneuvers reduce respiratory movements of the chest and abdomen to improve targeting accuracy and may reduce unintended vascular injury or capsular rupture and tumor spillage.

Ultrasound guidance (9–13, 20), coaxial technique (9, 11, 13, 20), general anesthesia or sedation (10–13) and needle biopsy under laparoscopic or video-assisted guidance in selected situations (10, 20) are described in published pediatric literature. In contrast, systematic Doppler mapping and respiratory holds reflect our institutional practice and expert opinion.

As outlined above, liver tumor biopsy should be regarded as a high-risk intervention and thus should be performed only in highly specialized pediatric surgery centers with appropriate expertise. Strategies for the prevention and management of hemorrhagic complications must be established before the procedure is initiated. In suspected high-risk tumors, blood products should be immediately available, pediatric intensive care units should be informed, and interventional radiology or hepatobiliary surgery backup should be defined. Persistent bleeding should prompt early imaging and consideration of angiography with selective embolization.

Based on our experience, the available literature and existing study protocols, we propose a stepwise safety framework (Figure 1). First, every child with suspected malignant liver tumor should undergo high-quality imaging followed by review in a multidisciplinary tumor board. Second, the team should decide whether tissue is required or if initiation of treatment is possible without biopsy, whether this consists of upfront resection or initiation of chemotherapy or other remedies, such as propranolol in the case of certain vascular lesions. Third, if biopsy is indicated, tumor rupture or active bleeding should be excluded; if present, hemodynamic stabilization takes priority (also when found intraoperatively) and the biopsy is individualized and performed in a second step. Fourth, if biopsy is indicated, image-guided core needle biopsy should be the default approach whenever feasible. Fifth, the biopsy should be performed under pediatric general anesthesia with controlled ventilation, Doppler-guided trajectory planning and coaxial technique when appropriate visualization, including laparoscopy if necessary. Finally, every institution performing these procedures should have a defined post-biopsy monitoring and hemorrhage escalation pathway.

This report has several limitations. It describes three consecutive referrals rather than a large institutional cohort. The cases were referred after complications had occurred and information from outside hospitals was incomplete for some variables, including exact operative timing, total transfusion volumes, and long-term oncologic outcomes. The manuscript therefore cannot prove that image-guided core needle biopsy, which is not risk free, would have prevented these complications. As in any narrative review, the literature was selected non-systematically and may be subject to selection bias, and some technical recommendations reflect institutional practice. However, the cases demonstrate the severity of complications after open surgical biopsy or manipulation. Furthermore, they are consistent with up-to-date available pediatric HB biopsy data supporting percutaneous biopsy when feasible (11, 14, 17).

## Conclusion

Open surgical biopsy in pediatric liver tumors can result in severe and potentially catastrophic, hemorrhagic complications. In these three consecutive patients, hemorrhage and coagulopathy required packing, temporary abdominal closure, transfusion and intensive care transfer and, in one patient, led to acute liver and kidney failure. These events underscore that liver tumor biopsy in children should be carefully planned and seen as a high-risk multidisciplinary procedure. When histological confirmation is required, image-guided core needle biopsy should be considered the preferred strategy whenever feasible. Open biopsy remains an option in selected situations, such as unsafe or inadequate percutaneous access, in specialized centers with immediate access to pediatric hepatobiliary surgery, interventional radiology and intensive care specialists.
